# *In vitro* antimicrobial effect of different root canal sealers against oral pathogens

**DOI:** 10.29252/cmm.3.2.7

**Published:** 2017-06

**Authors:** A Monajemzadeh, S Ahmadi Asoor, S Aslani, B Sadeghi-Nejad

**Affiliations:** 1 Department of Endodontics, School of Dental Medicine, Ahvaz Jundishapur University of Medical Sciences, Iran; 2 Department of Endodontics*,* School of Dental Medicine, Ahvaz Jundishapur University of Medical Sciences, Arvand International Division, Iran; 3 Student Research Committee, Kerman University of Medical Sciences, Kerman, Iran; 4 Abadan School of Medical Sciences, Abadan, Iran

**Keywords:** Antimicrobial, Epoxy resin AH-26, Mineral trioxide aggregate (MTA), Zinc oxide-eugenol cement

## Abstract

**Background and Purpose:**

Root canal therapy is the primary method for the treatment of an infected pulp in modern dentistry. The main aim of endodontic treatment is the elimination of bacteria and their products from infected root canals. In this study, we attempted to investigate the antimicrobial activity of three root canal sealers against oral pathogens.

**Materials and Methods:**

The antimicrobial effectiveness of three endodontic sealers with different chemical compositions, namely resin (AH 26), zinc oxide eugenol (ZOE), and mineral trioxide aggregate (MTA), against* Candida albican*s,* Streptococcus sanguis, Streptococcus salivarius, Streptococcus mutans*, and* Lactobacillus casei *was assayed by agar well diffusion method (AWDM). The tested sealers were prepared according to the manufacturer’s instructions and poured in the prepared wells of agar plates; diluted inocula (10^5^ and 10^6^ CFU/ml) of the tested microorganism strains were also used. The minimum inhibitory concentration (MIC) values of the selected canal sealers ranged between 3.12 and 50 mg.ml^-1^ against the employed microorganism strains. All the plates were incubated at 37°C under anaerobic condition for bacteria and at 30°C for *C. albicans. *After three days, the inhibition zones were measured.

**Results:**

In this investigation, AH 26 exhibited strong activity against *C. albicans* with the minimum inhibitory concentration of 12.5 mg.ml^-1^, but ZOE and MTA did not act against *C. albicans*. ZOE sealer had the highest antimicrobial activity against the tested bacteria, while MTA showed the lowest antimicrobial activity.

**Conclusion:**

The ascending sequence of microbial growth inhibition zones was as follows AH 26 > ZOE > MTA.

## Introduction

Microbes and their products are the most common etiologic agents of pulpitis and apical periodontitis [1-2]. Ercan et al. investigated microorganisms of infected dental root canals in 197 cultivable isolates and reported *Streptococcus *spp. (14.2%), *E. faecalis* (9.6%), *S. salivarius* (8.6%), *Lactobacillus* spp. (7.1%), *Actinomyces* spp. (7.1%), *Candida albicans* (4.1%), *Bacillus* spp. (2.0%), and *Escherichia coli* (1.6%) [3]. One of the main causes of root canal treatment failure is the presence of facultative and resistant microbial species of the oral cavity, namely *C. albicans, Enterococcus faecalis*, *Staphylococcus aureus*, and *Streptococcus* spp. [4]. 

*C. albicans *is a polymorphic fungus and a normal oral flora, which is in the oral cavity of up to 75% of the population and resides as a lifelong and harmless commensal agent. Several factors and activities have been recognized to contribute to the pathogenic potential of this fungus. Among them, the secretion of hydrolytic enzymes, molecules that cause adhesion and attack host cells, yeast-to-hypha mutation, biofilm formation, and phenotypic switching are considered the virulence factors of this fungus [3, 5]. Root canal sealers help minimize leakage, provide antimicrobial activity to reduce the possibility of residual bacteria, and resolve periapical lesions. The main objective of endodontic treatment is to omit microbes from the root canal and suppress them from infecting or re-infecting the root canal or the periapical tissues [6]. 

Thorough removal of microorganisms from the root canal system in all patients is not possible; therefore, the use of root canal filling materials with antimicrobial activity is considered for reducing microorganisms and preventing infection. Several studies have been performed in the recent years to recognize the antimicrobial efficacy of different endodontic sealers [7-16]. There is some evidence as to the antimicrobial activity of root canal sealers such as mineral trioxide aggregate (MTA), zinc oxide eugenol (ZOE), and resin (AH 26). MTA was first presented to endodontics by Torabinejad et al., and it has been used successfully for repairing root and focal perforations [17]. MTA has been expanded to seal the communication passages between the root canal system and the external surface of the tooth. Previous studies reflected that MTA is capable of stimulating antimicrobial activity and it has good sealing ability [18-19]. Numerous studies have evaluated the antimicrobial activities of endodontic sealers by agar diffusion method [7, 9, 15-16]. ZOE has shown antimicrobial activity because the ZOE components can inhibit growth of microorganisms in agar culture medium [15]. 

In this study, we investigated the effects of different root canal sealers including AH 26, ZOE, and MTA on five types of isolated oral pathogenic microorganisms (i.e., *C. albicans, S. sanguis, S. salivarius, S. mutans*, and* L. casei*)*.*

## Materials and Methods

***Endodontic sealers***

In the current study, the selected root canal sealers were AH 26 (Dentsply De Trey, Konstanz, Germany), ZOE (Kemdent Work Ltd, England), and MTA (Angelus, Londrina, PR, Brazil).

***Microbial isolation***

The selected anaerobic bacteria were *S. sanguis, S. salivarius, S. mutans, L. casei*, and* C. albicans*. For the antibacterial assays, all the bacterial samples were prepared from frozen stock cultures and were stored at -80°C in Trypticase Soy Broth (TSB) (Difco Labo ratories, Detroit, Mich., USA) complemented with 15% glycerol [9]. This medium was acquired from the Department of Medical Microbiology, Ahvaz Jundishapur University of Medical Sciences, Ahvaz, Iran. Then, these samples were subcultured in blood agar, incubated at 37ºC, and supplemented with 5% CO_2_ for 48 h before evaluation.

*C. albicans* isolates were obtained from the patients with periodontitis and gingivitis visiting the educational clinics of School of Dentistry, Ahvaz Jundishapur University of Medical Sciences, Ahvaz, Iran. The samples were previously identified based on colony color on CHROMagar *Candida* medium (CHROMagar, France). The phenotypic identification included germ tube formation at 37°C in bovine serum, chlamydoconidia formation on corn meal agar medium (Merck, Germany) plus 1% Tween 80, and evaluation of the growth ability of *C. albicans* at 45°C, which is in accordance with the instructions provided in previous studies [20, 21]. Stock fungal strains were subcultured on Sabouraud Dextrose Agar (SDA; Merck, Germany) and were maintained at 4°C until testing was performed.

***Antimicrobial activity***

*In vitro* antimicrobial efficacy of different root canal sealers was evaluated by AWDM. AWDM was performed in accordance with the descriptions of Shialy et al. [22]. A loop of cells from the freshly grown stock cultures was removed to the test tubes of nutrient broth medium (NBM) for bacteria, and Sabouraud dextrose broth (SDB) was used for *Candida *spp.; the media were incubated overnight at 37°C for 18-24 h. Subsequently, the cultures were diluted in sterile normal saline solution (0.9%) to obtain 5 × 10^5 ^spore/ml for fungal strains and 10^6^ CFU/ml for the bacterial strains standardized with the turbidity of 0.5 McFarland [23]. 

Microbial inoculation was performed using sterile cotton swab sticks, and five wells with 6 mm width (diameter) were punched in each agar plate. Then, 100 µl aliquots of freshly prepared sealers were placed in the wells immediately after mixing. The plates were maintained at room temperature for 2 h to allow prediffusion of the materials. 

Agar plates inoculated with bacteria were placed in an anaerobic cabinet supplied with CO_2_ at 37°C for 24 h, 48 h, and 72 h, and agar plates were inoculated with *C. albicans* and incubated at 30°C for 2-3 days [24]. Positive control plates were streaked with bacteria, but no root canal sealer was applied. The diameters of inhibition zones around the wells were measured with a millimeter (mm) ruler with the accuracy of 0.5 mm and recorded for each sealer. All the assays were performed in triplicate. 

***Statistical analysis***

The data were analyzed using Kruskal-Wallis test in SPSS, version 20. *P-values* less than 0.05 were considered statistically significant.

## Results

**Figure 1:**
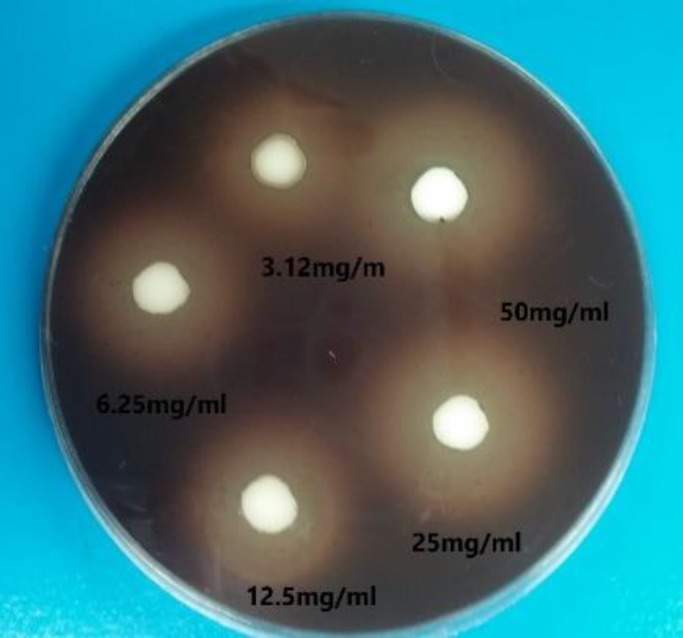
Inhibition zones increased by zinc oxide eugenol against S. salivarious on blood agar

**Figure 2:**
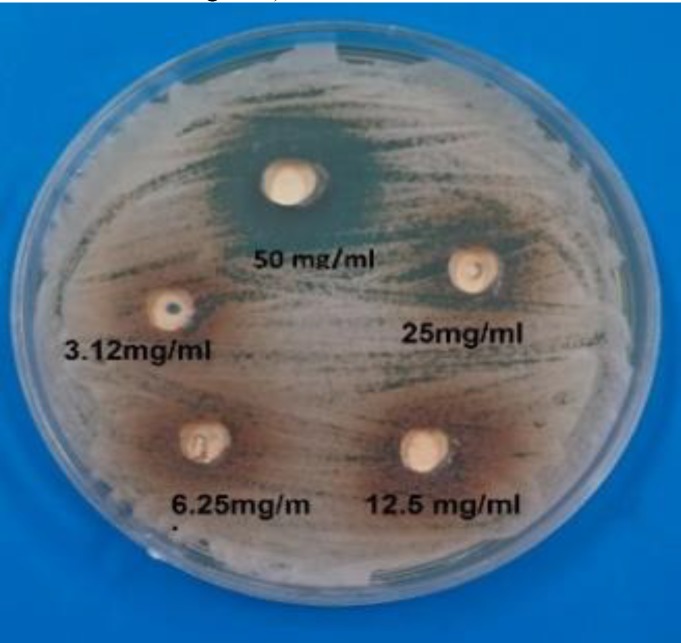
Inhibition zones increased by resin sealer against C. albicans on Sabouraud dextrose agar

The antimicrobial efficacy of each endodontic sealer was measured by the diameter of the inhibition zone around each well filled by a sealer for each kind of bacterium and fungus. AH 26 exhibited a large inhibition zone (26 mm) against *C. albicans *with minimum inhibitory concentration (MIC)=12.5 mg.ml^-1^, but ZOE and MTA did not affect *C. albicans*. AH 26 was not efficient against *S. sanguis *and *S. salivarius, *but it was effective against *S. mutans *and* L. casei*, and it exhibited strong efficacy against *L. casei* with a large inhibition zone (30 mm) and MIC=3.12 mg.ml^-1^. MTA revealed moderate antimicrobial activity against* S. sanguis, S. salivarius,* and* S. mutans *(range of inhibition zones 13-28 mm and MICs=12.5-50 mg.ml^-1^), but it did not show antimicrobial activity against* L. casei*. ZOE had strong antibacterial efficacy against *S. sanguis, S. salivarius, S. mutans, *and *L. casei* with inhibition zones of 15-28 mm and MICs of 50-3.12 mg.ml^-1 ^(Figures 1 and 2; Table 1). The *P-values* for each sealer against all the tested strainsare presented in Table 2. Table 3 demonstrates the results of paired comparison of the sealers. 

**Table 1:** Antimicrobial activity (mm inhibition zone diameter) of three root canal sealers on the selected microorganisms

**Root canal sealers**															**Microorganisms**
**3.12 mg.ml** ^- 1^			**6.25 mg.ml** ^- 1^			**12.5 mg.ml** ^- 1^			**25 mg.ml** ^-1^			**50 mg.ml** ^- 1^			
**AH 26**	**MTA**	**ZOE**	**AH 26**	**MTA**	**ZOE**	**AH 26**	**MTA**	**ZOE**	**AH 26**	**MTA**	**ZOE**	**AH 26**	**MTA**	**ZOE**	
0	0	0	0	0	0	15	0	0	20	0	0	26	0	0	*Candida albicans*
0	0	15	0	0	20	0	0	22	0	7	25	0	8	27	*Streptococcus sanguis*
0	0	15	0	0	20	0	8	22	0	12	24	0	13	25	*Streptococcus salivarius*
0	0	13	0	0	15	15	0	18	18	12	20	20	13	25	*Streptococcus* *mutans*
18	0	20	20	0	24	22	0	25	25	0	27	30	0	28	*Lactobacillus casei*

Abbreviations: Mineral trioxide aggregate (MTA); resin (AH 26); zinc oxide eugenol (ZOE)

**Table 2:** Statistical analysis of one sample t-test

**Root canal sealers**	**Mean±SD**	**Significant (2-tailed)**	
Zinc oxide eugenol	18.2±7.84	0.001	*P<0.05*
Mineral trioxide aggregate	6.92±3.63	0.001	*P<0.001*
Resin	3.80±8.65	0.001	*P<0.05*

**Table 3:** Paired-samples t-test for comparison of means

**Root canal sealers**	**Mean±SD**	**Significant (2-tailed)**	
Pair 1 AH 26-MTA	6.88±8.97	0.001	*P<0.05*
Pair 2 ZOE - MTA	11.28±7.032	0.001	*P<0.001*
Pair 3 ZOE – AH 26	4.40±10.054	0.039	*P<0.05*

Abbreviations: Mineral Trioxide Aggregate (MTA); Resin (AH 26); ZOE (Zinc Oxide Eugenol)

Significance level < 0.05

## Discussion

In this study, we investigated the antimicrobial activity of three different sealers including ZOE, MTA, and AH 26 against *C. albicans, S. sanguis, S. salivarius, S. mutans, *and *L. casei *by AWDM, which is most commonly used for the assessment of antimicrobial activity. This method permits drawing direct comparisons between materials and demonstrates which sealers are more probable to have antimicrobial activity within the root canal system [25]. The antimicrobial activity of root-canal sealers may be an initial factor in preventing the regrowth of microorganisms and control of microbial return into the root canal system. Since the antimicrobial ingredients in the root-canal sealers do not have selective toxicity against microorganisms, they may also show toxic effects on host cells [26]. According to the current study, AH 26 sealer with the mean growth of 18.2 mm and MTA sealer with mean growth of 6.92 mm had the highest antimicrobial effects. Our results were in accordance with those of Shantiaee and Dianat, Mohammadi and Yazdizadeh, Tabrizizadeh and Mohammadi, and Al-Khatib et al*. *studies, which used similar methodologies and found that AH 26 had the largest inhibition zone in comparison with the other tested sealers [9, 27-29]. Shantiaee et al. evaluated antimicrobial efficacy of three root-canal sealers, namely AH 26, calcium hydroxide (Apexit), and ZOE, and observed that the antibacterial activity of AH 26 was significantly greater than the other tested materials. ZOE sealer had a moderate effect on the tested microorganisms, whilst Apexit had the lowest antibacterial effect on *Streptococcus mutans *and no antibacterial activity against *Prevotella melaninogenicus *[9]. In addition, Ehsani et al. reported that AH 26 sealer had the highest antibacterial activity against *E. faecalis* and *L. casei *[30]*.* This finding is similar to our result in that the inhibition zone of AH 26 sealer against *L. casei *showed the largest measure (30 mm) in comparison with ZOE and MTA sealers (Table 1). Inversely, other experimental evidence suggested that AH 26 had the lowest or no antimicrobial activity [12, 14, 31, 32].

Further, our results revealed development of the inhibition zone of AH 26 after three days. This finding was in agreement with those of other studies since AH 26 sealer had ideal antimicrobial properties after 72 hours and the inhibition zone declined afterwards [33]. Antimicrobial properties of resin-based sealers such as AH 26 may be attributed to the formaldehyde release in the polymerization process [15, 25, 34, 35]. Additionally, when ZOE is applied to a dentinal cavity, the small quantities of eugenol diffuse through the dentin to the pulp. Low concentrations of ZOE have anti-inflammatory and local anesthetic effects on the dental pulp. Thus, the use of ZOE temporary filling may facilitate pulpal healing, while high eugenol concentrations are cytotoxic. [36]. MTA sealer contains calcium oxide, which forms calcium hydroxide in contact with water and confers antibacterial property to MTA [11, 18, 33]. Also, the antimicrobial activity of MTA was reported by Torabinejad et al., [18] who reported its efficiency against a few facultative bacteria, but no efficacy was detected against *C. albicans, E. faecalis*, *Staphylococcus aureus*, *Bacillus subtilis*, *Escherichia coli*, or anaerobic bacteria, while in our study, MTA was active against anaerobic bacteria such as *S. sanguis*, *S. salivarious*, and *S. mutans*. Moreover, it was inactive against *L. casei *and* C. albicans* similar to the above-mentioned findings [18]*.*

Stowe et al. [19] evaluated the antimicrobial activity of MTA and reported that it inhibited the growth of *E. faecalis *and *S. sanguis. *Our results were in consonance with those of Stowe et al. [19], who reported the activity of MTA sealer against *S. sanguis. *In addition, Al-Nazhua and Al- Judal [37] stated that MTA at a concentration of 50 mg.ml^-1^ inhibited the growth of *C. albicans* after the third day, while MTA in our study did not reveal any activity against *C. albicans*. Diversity in the employed microbial strains and the testing methods may be the main reasons for these discrepancies. Also, Siquera suggested that different survey methods (e.g., AWDM) may be the main reason for the incongruence between our findings and those of other studies [25]. 

On the other hand, previous studies reported that MTA decreases the percentage of fibroblasts and macrophages in the synthesis phase of DNA (the major event in S-phase is DNA replication) and increases their cytotoxicity [38]. In the present study, ZOE had strong antibacterial effect against the tested bacteria because ZOE components can diffuse through the agar [15]. In addition, the antimicrobial efficacy of ZOE has been associated with free eugenol released from the material [11]. ZOE sealer, as a phenolic composition, acts against mycotic cells and other microorganisms by protein denaturation where the protein becomes non-loyal [9, 11, 25, 35, 39]. The current findings also revealed that the inhibition zone of ZOE in facultative anaerobic bacteria such as *S. mutans* increased after the third day, although it reduced in *C. albicans* during the same time interval. 

It seems that ZOE is more suitable for limitation of facultative anaerobic bacteria. The results of the present study were in accordance with those of the previous ones performed by Markowitz et al. [36] and Saggar et al. [40], demonstrating that ZOE sealer was more efficient in inhibiting microorganisms. On the other hand, a number of studies reported cytotoxicity of the eugenol component of ZOE [41, 42]. If eugenol is put in contact with the oral soft tissue, it can cause tissue signs, allergic reaction, and contact dermatitis/stomatitis [43, 44]. Just like most sealers, AH 26 is highly toxic when freshly prepared. However, this toxicity decreases rapidly during infixing, and after 24 h, the cement changes to one of the least toxic endodontic sealers [45]. Although MTA sealer has some merits such as high alkalinity (bacteriostatic), hydrophilia, radioopacity, low solubility, and excellent sealing ability (low marginal leakage), it has a number of disadvantages such as cost-intensiveness, irreversible usage, and difficult retouch [46-48]. 

An ideal root-canal sealer should be able to kill the microorganisms present on the dentinal walls of root canals and those present deep inside the dentinal root canals; they should also have low toxicity for the surrounding tissues [49]. The root-canal sealers should not only kill microorganisms on contact, but also they should be able to diffuse inside the dentinal root canals, which is possible only if the sealer has good flow properties. Finally, endodontic sealers that possess suitable flowability and antimicrobial properties may aid in the elimination of microorganisms located in the root canal system [25]. Comparison of the sealers revealed that AH 26 and ZOE showed significantly higher antimicrobial activity as compared to the MTA sealer. In addition, AH 26 revealed significantly higher antimicrobial activity relative to ZOE. In summary, the ascending sequence of microbial growth inhibition zones was as follows AH 26 > ZOE > MTA.

## Conclusion

We found no anti-candida activity in different concentrations of ZOE and MTA sealers, whereas AH 26 showed strong anti-candida activity against *C. albicans*. ZOE and MTA presented strong and moderate anti-bacterial activities against *S. sanguis *and *S. salivarious*, respectively**, **but AH 26 did not show anti-bacterial activity against *S. sanguis *and *S. salivarious*. Overall, AH 26 sealer had the greatest antibacterial activity, while MTA had the lowest.
